# Dermatophytosis caused by *Trichophyton mentagrophytes* complex in organic pigs

**DOI:** 10.1186/s13028-023-00695-w

**Published:** 2023-07-11

**Authors:** Kaisa Ryytty Sylvén, Ann-Louise Bergefur, Magdalena Jacobson, Per Wallgren, Lena Eliasson Selling

**Affiliations:** 1 Farm & Animal Health Sweden, Gård och Djurhälsan AB, Uppsala, Kungsängens gård 731 43 Sweden; 2 Department of Microbiology, National Veterinary Institute (SVA), Uppsala, 751 89 Sweden; 3 Department of Clinical Sciences, Swedish University of Agricultural Sciences (SLU), Box 7054, Uppsala, 750 07 Sweden; 4 Department of Animal Health and Antimicrobial Strategies, National Veterinary Institute (SVA), Uppsala, 751 89 Sweden

**Keywords:** Dermatophytosis, Ringworm, Skin disease

## Abstract

**Background:**

Dermatophytosis (ringworm) caused by members of the *Trichophyton mentagrophytes* complex is rarely diagnosed in pigs but has been recognized as an increasingly common infection in humans. Further, resistance to antifungal drugs have been reported both in Asia and in Europe. This is the first scientific report of infection by the *T. mentagrophytes* complex in pigs in the Nordic countries.

**Case presentation:**

Skin lesions developed in grower pigs in an organic fattening pig farm with outdoor production and following laboratory analyses, dermatophytosis caused by members of the *T. mentagrophytes* complex was diagnosed. Infection was linked to poor hygiene, high humidity, and moderate outdoor temperatures, in combination with high pig density. A farm worker developed a skin lesion after close contact with affected pigs, which highlighted the zoonotic potential of porcine dermatophytosis. The dermatophytes may have originated from the herd supplying the growers where similar lesions occurred in pigs. Further, pigs from another organic fattening herd that received growers from the same supplier herd also developed dermatophytosis. The lesions healed without treatment as the housing conditions were improved. Isolation of affected pigs prevented spread to other pigs

**Conclusion:**

Members of the *T. mentagrophytes* complex can cause ringworm in pigs. The fungi probably persist in the haircoat and may cause overt disease when environmental conditions promote growth of mycelia.

## Background

Dermatophytosis (ringworm) in pigs may be caused by several fungal species having a varying zoonotic potential [1]. Members of the *Trichophyton mentagrophytes* complex are frequently isolated from humans, and in recent years, antifungal drug resistance has been reported from Asia and Europe [2–5]. All mammals are considered susceptible to *T. mentagrophytes.* Rodents are an important reservoir for *T. mentagrophytes*, and the fungus is also often isolated from various hosts such as carnivores, horses, and rabbits [1, 6]. In pigs, however, only a few scientific reports are available [7–9]. The incubation period in humans is 4–10 days, whereas in animals, it has been reported to vary between 1 and 3 weeks [10]. In most animal species, ringworm caused by *T. mentagrophytes* produces ring-like lesions with a diameter of up to 20 cm [10, 11]. However, in all age categories of pigs, *T. mentagrophytes* generates red, orange patches with a brownish dry crust [8, 9, 11].

The fungal spores of *T. mentagrophytes* may survive for years in cool and dry environments [11–14]. Poor hygiene and high stocking densities, high humidity and moderate environmental temperatures favor the development of lesions [10]. Other risk factors include malnutrition and an immunocompromised host state [1, 2]. As rodents may carry fungi, preventive measures consequently include rodent control, proper waste management, and covering of open straw stacks and feed stores [1, 12]. Prevention also includes preventing contacts between susceptible species, such as those mentioned above, and cleaning and disinfection of premises [1, 2, 12, 15].

Dermatophytes adhere to keratinocytes by adhesins and other mechanisms [14]. The fungi produce enzymes, such as proteases, that dissolve keratin and utilize degradation products as nutrients [16–18]. Penetration of the *stratum corneum* may be facilitated by high humidity, which moistens and loosens the barrier function, or by injuries to the skin [2, 16–18]. Infection is generally limited to the *stratum corneum* since fungi are not able to penetrate the deeper layers of the skin in immunocompetent hosts [16–18]. Lesions of *T. mentagrophytes* generally resolve within ten weeks without treatment [10]. However, two different treatment strategies may be applied; either crust removal followed by locally applied antifungal products, or oral medication with fungicides [5, 12, 19, 20].

To reach a diagnosis, it is essential to obtain a complete medical history and perform a thorough clinical examination with characterization of the lesions [15]. Traditional methods for identification of fungi include cultivation and microscopy of skin scrapings or biopsies, to distinguish their characteristic macroscopic and microscopic morphology [2, 21–26]. More recently, MALDI-TOF MS (Matrix Assisted Laser Desorption – Time of Flight Mass Spectrometry), has been used to obtain a diagnosis [27].

The aim of this case report was to describe the diagnosis and the clinical findings caused by members of the *T. mentagrophytes* complex in a pig herd that reared fatteners outdoors. In addition, the zoonotic aspect, the epidemiology, and the treatment possibilities were scrutinised.

## Case presentation

**Fig. 1:**
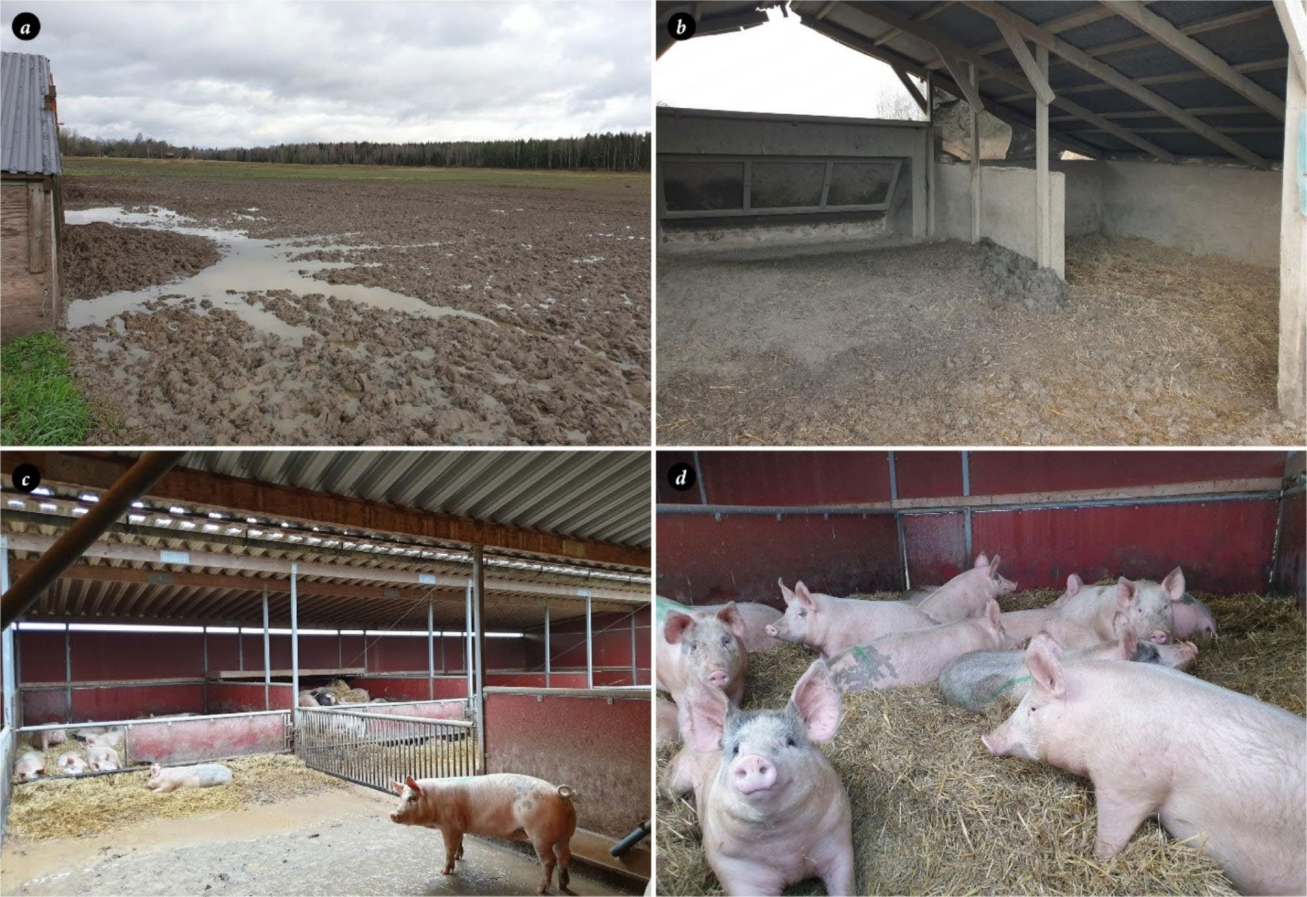
Housing of the pigs **a, b**) Pasture and huts one week after removal of pigs to the semi-indoor stable. Each pasture had three huts. Dry feed was served *ad libitum* in troughs and lying areas provided with straw were situated along the long-side walls. Lying area of 0.45 m^2^ per pig. **c, d)** semi- indoor stable with straw bedding, lying area of 2.2 m^2^ per pig

The farm was an organic fattening farm practicing partial outdoor rearing. In August 2021, the farm received 200, 12-week-old pigs with a body weight of 35 kg. The pigs were placed in a two-hectare pasture equipped with three wooden huts of 30 m^2^ each that provided a lying area of 0.45 m^2^ per pig. The pigs had access to an additional outdoor lying area which was bedded with silage (Fig. 1a). The farm had a grazing system with a rotational time of six years for each pasture. The pigs were fed dry feed *ad libitum* in troughs inside the huts (Fig. 1b).

**Fig. 2:**
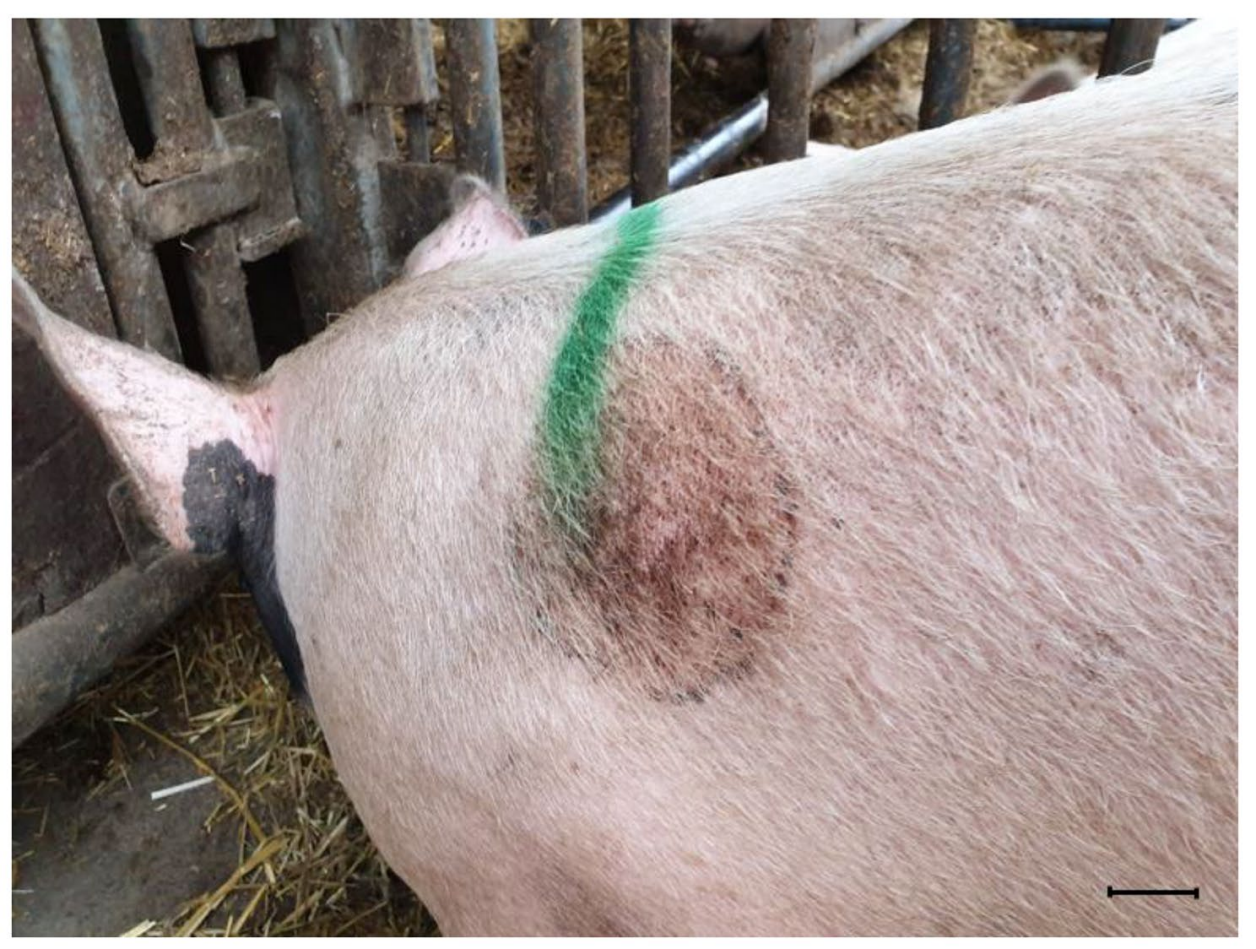
Skin lesion of pig Skin lesion of an affected pig at the initial examination nine weeks after arrival to the finishing farm. The lesion was circular, light red and covered with brownish material, with a distinct ring on the outer rim and had an oily appearance due to flakes of keratinized skin mixed with sebum. Discolored hairs could also be seen in the lesion. Bar = 4 cm

Approximately nine weeks after arrival, the pigs were moved to semi-indoor pens with straw beds, and with access to an outdoor area with a solid concrete floor (Fig. 1c, d). The lying area in these facilities was 2.2 m^2^ per pig. The pigs were fed with a liquid feeding system. One week later, the pigs were weighed. The mean daily weight gain (DWG) was calculated to approximately 850 g, which corresponded to the average DWG in the herd [28]. During weighing, the farmer noted single skin lesions in 10% of the fatteners. The lesions were circular, with a diameter of 7 to 13 cm, and situated on the flank, neck, or thorax of the pigs. The lesions were light red and covered with brownish material, with a distinct ring on the outer rim. The lesions had an oily appearance due to flakes of keratinized skin mixed with sebum, and some discolored hairs (Fig. 2). No alopecia or pruritus were evident.

**Fig. 3:**
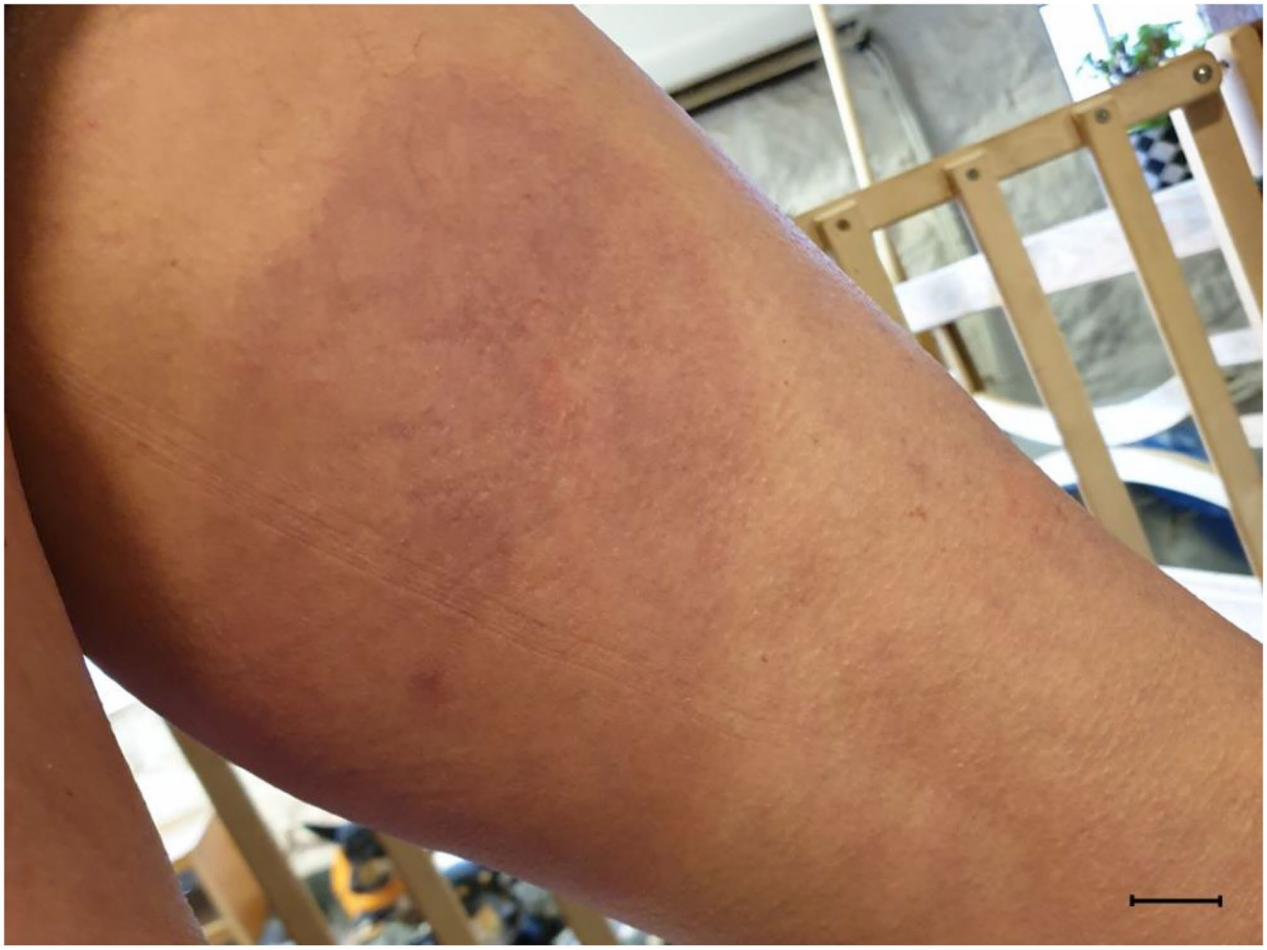
Skin lesion of farm worker Skin lesion of farm worker one week after handling the pigs at weighing. The lesion was circular and dark red in color. The farm worker did not experience any pruritus. Bar = 2 cm

Thirteen days later, the lesions had decreased in size and changed morphology. The affected skin area still appeared brownish and oily but was less red and appeared to be healing. At this time, one member of the staff that had weighed the pigs, had developed a skin lesion with a diameter of approximately 20 cm (Fig. 3). The person sought medical advice, but the skin lesion was not sampled.

Lesions of a similar morphology were also found in pigs at the grower supplier, as well as in another fattening herd that received growers from the same source. However, no samplings were performed in these herds.

**Fig. 4:**
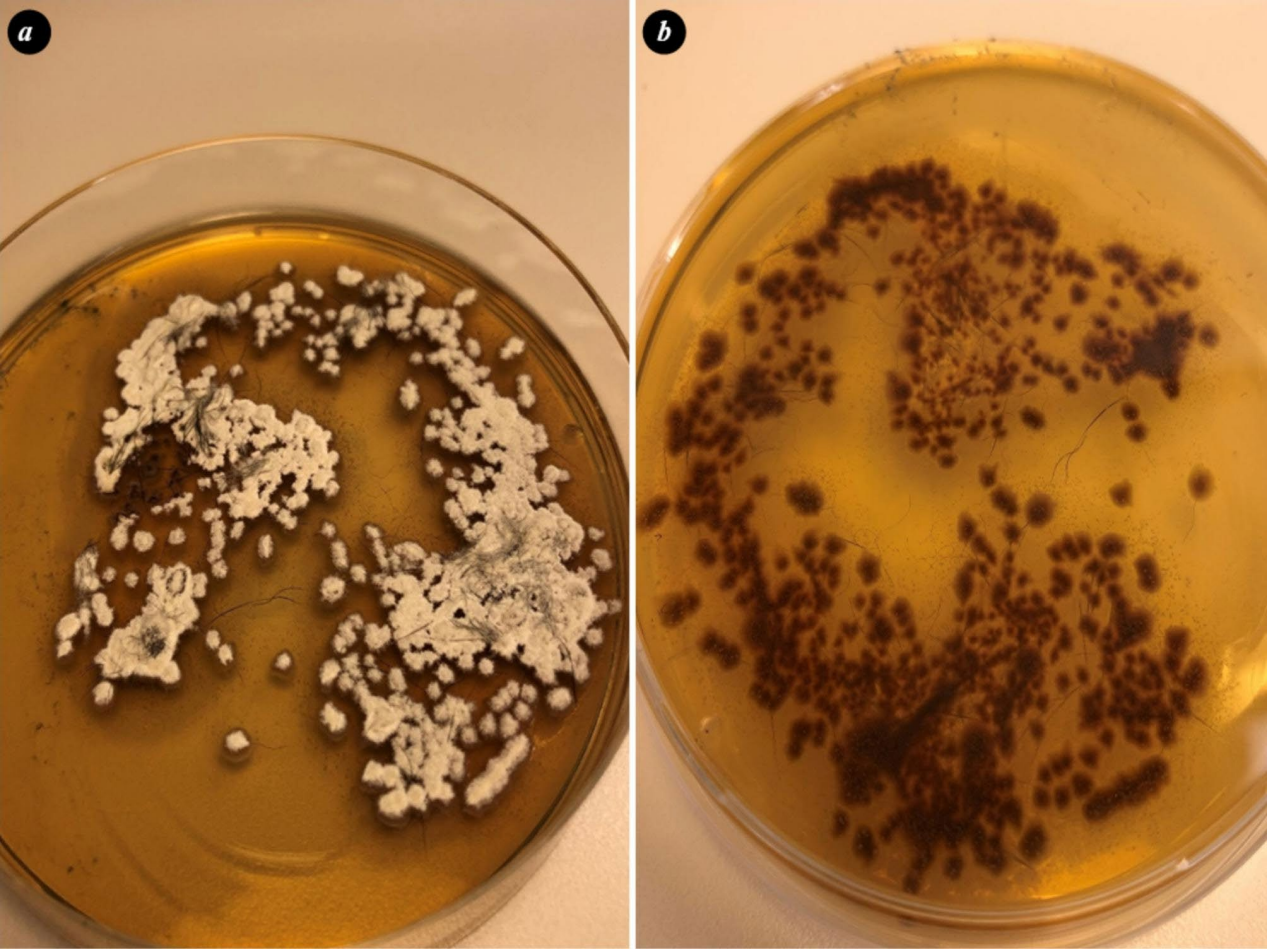
Dermatophyte (*T. Mentagrophytes* complex) growing on modified Dixon agar **(a)** The colonies flat, white in color, with a powdery appearance to the granular surface. **(b)** Reddish-brown reverse pigmention of the colonies growing on Dixon agar

**Fig. 5:**
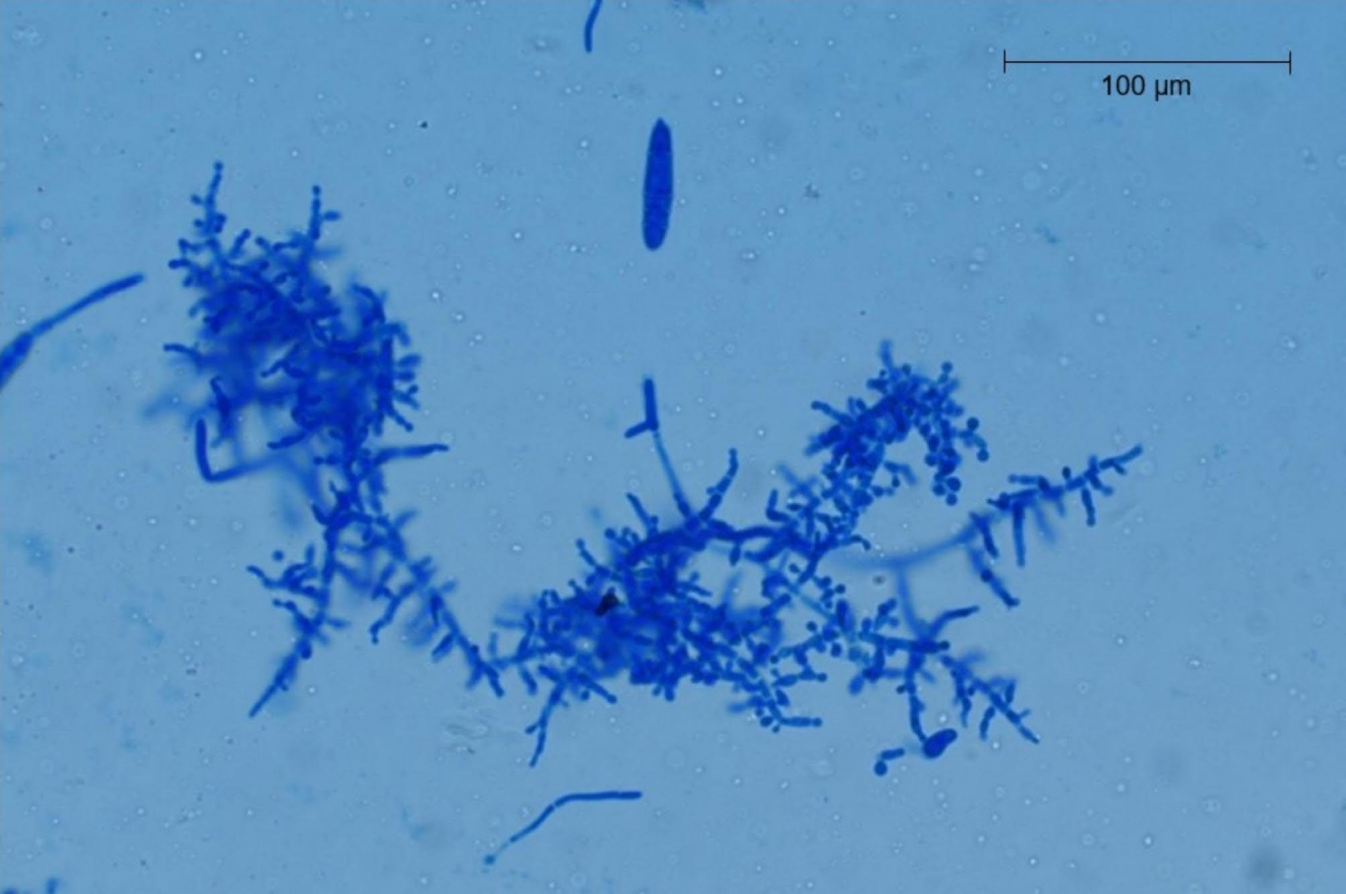
Microscopic image of the isolated dermatophyte (*T. Mentagrophytes* complex) *T. Mentagrophytes* complex isolated from a sampled pig, showing branched conidiophores bearing spherical microconidia in clusters extending from septate hyphae. One macroconidia seen in the middle upper part of the picture (Lactophenol Cotton Blue stain Obj x 40)

In the case herd, skin lesions from four pigs were sampled by scraping with a scalpel blade, plucking hairs, and brushing lesions with a toothbrush according to [29]. Fungal cultivation was performed at the Swedish National Veterinary Institute using selective agars. The samples were grown on modified Dixon agar with 2% salt followed by pure cultivation on Sabouraud´s dextrose agar (SAB). On Dixon agar the colonies were white in color, with a powdery appearance to the granular surface and a downy look. The colonies were pleomorphic, some flat and others with a raised central part (Fig. 4a). Reverse pigmentation was reddish-brown (Fig. 4b). On SAB agar, the dermatophyte appeared heaped and folded, buff to brown in color, with a suede-like surface texture and a characteristic, dark, reddish-brown submerged peripheral fringe, and reverse pigmentation. Tape-technique [30] was used for suspect colonies from SAB agar and colony material was stained with lactophenol cotton blue. Microscopy of the stained colony material showed microconidia (2–4 μm) which were spherical to pyriform in shape and the macroconidia (20–50 μm and 6–8 μm) were cigar- to club-shaped (Fig. 5). *T. mentagrophytes* complex was diagnosed in two pooled samples by the morphological appearance, including the presence of sessile microconidia in dense, grape-like clusters on the conidiophores according to [31].

At inspection of the huts and pasture nine weeks after arrival of the pigs, the pasture was muddy (Fig. 1a), the straw bedding in the huts was damp (Fig. 1b), and feed residues were found around the troughs. No rodents were observed, but the owner confirmed that rats were seen adjacent to the huts from time to time. During the nine-week period the pigs spent on pasture, the temperature varied from 6 to 19 ºC, with an average temperature of 12.5 ºC. A total of 150 mm of rain fell during the period, which was above average for the time of year [32]. The straw beds in the semi- indoor system, to where the pigs were moved after nine weeks on pasture and where the skin lesions were discovered, were dry but the feeding area was soiled with manure. A conservative treatment plan for the pigs was implemented. Affected pigs in the semi-indoor facilities were isolated in separate pens and hygiene measures were improved. Cleaning was initially restricted to the removal of the straw beds from the semi-indoor units. During the following pasture season, the semi-indoor units were washed with water and no pigs were kept in the units for four months. No disinfectants were used. The staff were recommended to wear gloves when handling the pigs and to wash their hands after working in the pig facilities.

Control measures aimed at reducing the risk of dermatophytosis also included changing pasture during next season, cleaning the huts and replacing all wooden materials, improving the straw bedding routines in the huts, lowering pig density per hut and implementing extended rodent control.

## Discussion and conclusions

Very few cases of dermatophytosis in pigs have been reported in the literature [7–9] and pigs seem to be relatively resistant to such infections. However, poor hygiene and adverse outdoor weather conditions in the autumn might have predisposed pigs in the case herd towards developing dermatophytosis. Most likely, the pigs developed skin lesions while at pasture, but these remained undetected until the individual handling at weighing. It is likely that the infection was already present in the pigs when they arrived at the fattening farm. Indeed, similar lesions were found in piglets in the grower-supplying farm, as well as at another organic fattening farm receiving growers from the same herd. Pigs may act as subclinical carriers of dermatophytes, and following the development of lesions, may transmit the infection to humans [1, 6, 17, 33].

The dermatophytes might also have been introduced by rodents as reported in [1, 6, 17] or possibly humans, or they could have been present in the environment, providing a reservoir for the infection. The adverse weather conditions with high humidity and moderate temperatures might have driven the pigs into the huts, causing crowding that, in turn, may have promoted spreading of *T. mentagrophytes*. Indeed, the hygiene in the huts was poor and the stocking densities were high, especially when the pigs had grown larger. Also, nutrient deficiency and immunosuppression may predispose to skin diseases [1, 2], but this hypothesis was considered less likely since the DWG was not affected.

In this case, topical treatment was not practically feasible for several reasons. For instance, topical treatment of dermatophytosis in outdoor-raised pigs under Swedish winter conditions can be challenging. Due to the risk of residues in meat products, the use of systemically distributed antifungal drugs in pigs is restricted, and no products are available for systemic use in Sweden [1, 34]. Thus, conservative treatment combined with improved hygiene measures was implemented. Lesions healed within six weeks, corresponding to what has previously been reported [7]. Morbidity was 10% within the affected group of pigs, but the average DWG was not affected. During the following nine months, no additional cases were identified.

The high survival rate of the dermatophyte arthroconidia in the environment and the multiplicity of host species, makes prevention difficult [35]. An optimal cleaning and disinfection protocol would have included cleaning with water and use of disinfectants effective against dermatophytes such as sodium hypochlorite or penta potassium-bis (peroximonosulfate)-bis (sulfate) [35, 36]. However, cleaning with water is not an option in uninsulated premises at temperatures below 0 ºC, as in Sweden during winter. Thus, cleaning was initially restricted to the removal of the straw beds from the units. For similar reasons, disinfection was not used, but as dermatophytosis was not diagnosed in the subsequent groups of pigs, the measures undertaken appeared to have been sufficient.

This case report describes a rarely diagnosed skin condition in pigs caused by members of the *T. mentagrophytes* complex, which has previously not been reported in the Nordic countries. The possibility of pigs being carriers of fungi without displaying clinical signs is discussed, and the zoonotic aspect highlighted. Challenges with treatment of the disease included lack of registered drugs towards the disease in pigs and the unsuitability of disease preventing measures such as washing and disinfection in outdoor facilities when the temperature is below 0 ºC.

## Data Availability

The laboratory reports are available from the corresponding author on reasonable request.

## Declarations

### Prior publication

Data have not been published previously.
